# Supplementary material for: Extensive disruption of protein interactions by genetic variants across the allele frequency spectrum in human populations
Source: Nat Commun. 2019 Sep 12;10:4141. doi: 10.1038/s41467-019-11959-3 (PMC6742646; doi:10.1038/s41467-019-11959-3)
Supplement: Supplementary file 1 — Supplementary Information [file 41467_2019_11959_MOESM1_ESM.pdf]

**Supplementary Information**

# Extensive disruption of protein interactions by genetic variants across the allele frequency spectrum in human populations

Robert Fragoza[1,2,*] , Jishnu Das[3,4,*], Shayne D. Wierbowski[1,2], Jin Liang[1,2], Tina N. Tran[5,6], Siqi Liang[1,2], Juan F. Beltran[1,2], Christen A. Rivera-Erick[1,2], Kaixiong Ye[1], Ting-Yi Wang[1,2], Li Yao[1,2], Matthew Mort[7], Peter D. Stenson[7], David N. Cooper[7], Xiaomu Wei[1], Alon Keinan[1], John C. Schimenti[5], Andrew G. Clark[1,6], and Haiyuan Yu[1,2,†]

1. Department of Computational Biology, Cornell University, Ithaca, NY 14853, USA
2. Weill Institute for Cell and Molecular Biology, Cornell University, Ithaca, NY 14853, USA
3. Ragon Institute of MGH, MIT and Harvard, Cambridge, MA 02139, USA
4. Department of Biological Engineering, Massachusetts Institute of Technology, Cambridge, MA 02139
5. Department of Biomedical Science, Cornell University, Ithaca, NY 14853, USA
6. Department of Molecular Biology and Genetics, Cornell University, Ithaca, NY 14853, USA
7. Institute of Medical Genetics, Cardiff University, Heath Park, Cardiff CF14 4XN, UK

†Correspondence should be addressed to H.Y. (haiyuan.yu@cornell.edu)
*These authors contributed equally to this work.

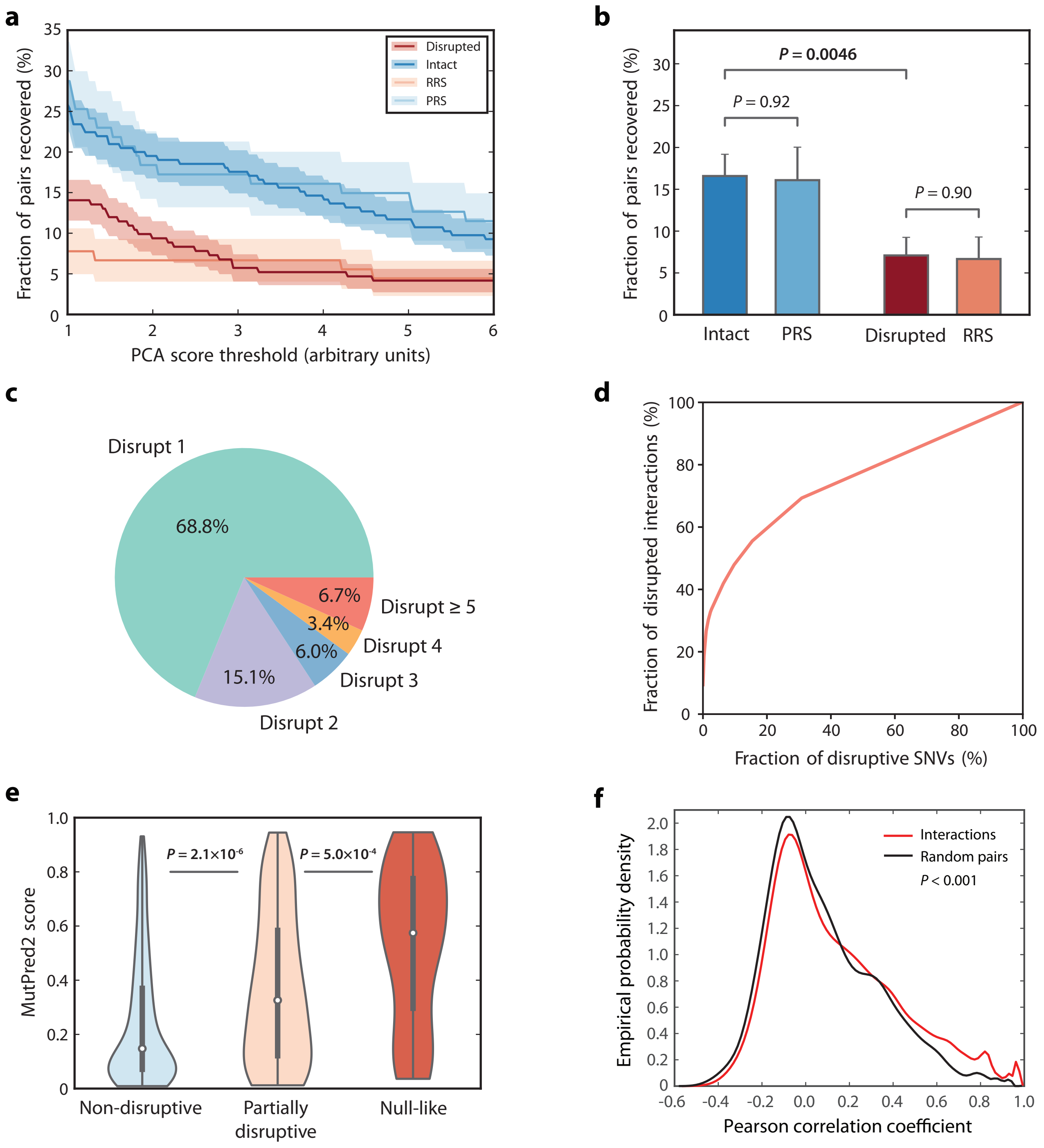


**Supplementary Figure 1**. Distribution and reproducibility of disrupted and non-disrupted SNV-interaction pairs. (**a**) Fraction of protein pairs recovered by PCA across increasingly stringent PCA scoring thresholds. SE of proportion is demarcated by shading. (**b**) Fraction of protein pairs recovered by PCA for disrupted and intact interactions in comparison to positive and random reference sets (PRS and RRS). Interactions corresponding to SNVs found on overrepresented bait proteins (bait has >20 interaction partners listed in Supplementary Data 2) were removed. *P* values by one-tailed *Z*-test between disrupted and intact interactions. *P* values by two-tailed *Z*-test for all other comparisons. (**c**) Fraction of disruptive variants ($n$ = 298) categorized by number of disrupted interaction partners. (**d**) Cumulative distribution function plotting the fraction of disruptive variants against the total fraction of interactions perturbed. (**e**) Distribution of MutPred2 scores across three disruption categories. Thick black bars are the interquartile range, white dots display the median, and extended thin black lines represent 95% confidence intervals. *P* values by one-tailed *U*-test. (**f**) Co-expression of protein abundance levels for protein interaction pairs used in this study. Interacting protein pairs were significantly more likely to be co-expressed than random protein pairs in tissue and cell data from the Human Proteome Map. *P* value by two-sided *KS* test. See also Supplementary Note 1.

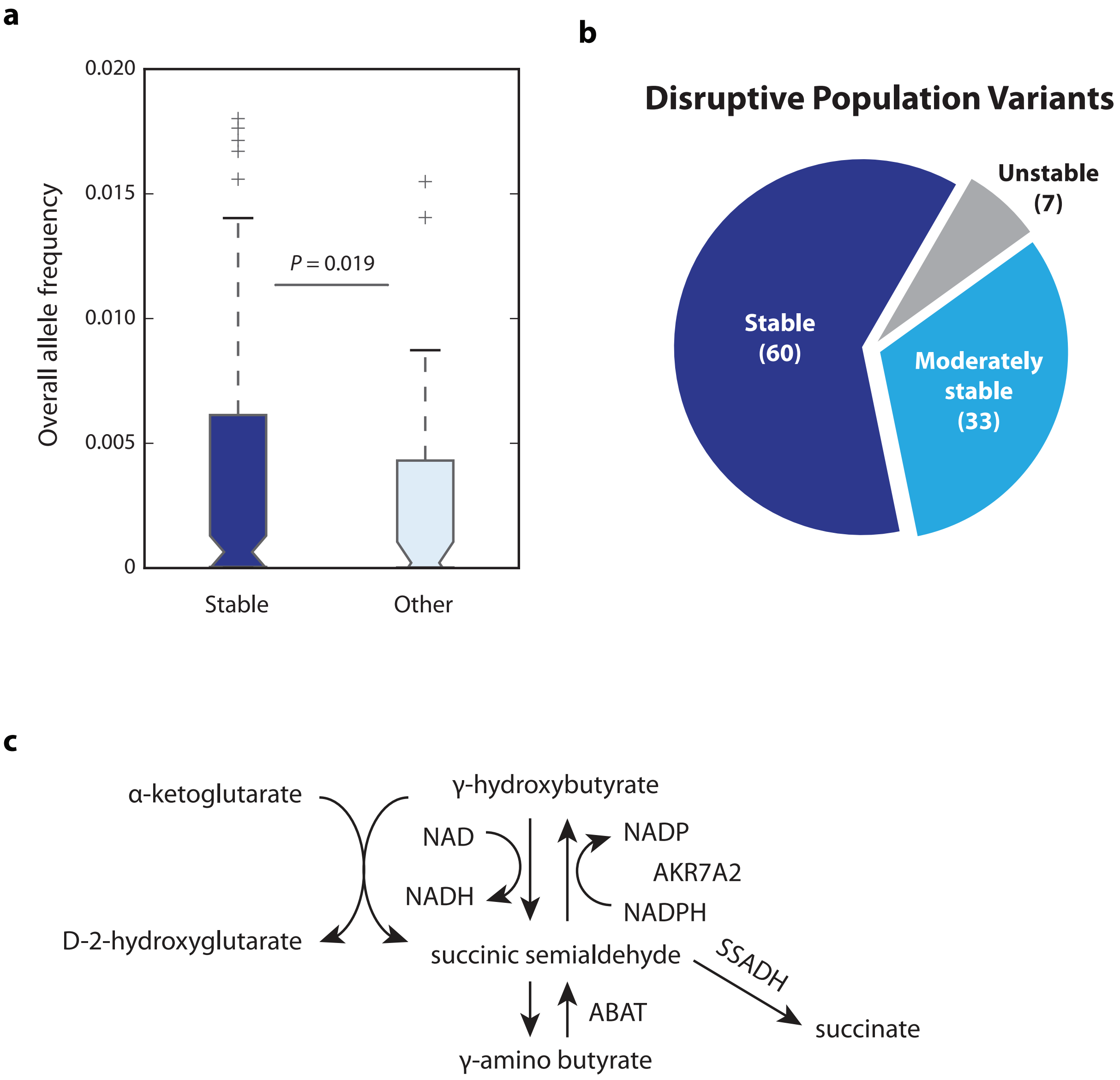


**Supplementary Figure 2**. Protein-destabilizing variants are selectively constrained and do not fully account for interaction perturbation phenotypes. (**a**) Distribution of allele frequencies for variants categorized as stable ($n$ = 214) or other ($n$ = 64). Other was constructed by combining moderately stable and unstable variants. $P$ values by one-tailed $U$-test. (**b**) Distribution of interaction-disruptive ExAC variants across three stability categories. (**c**) γ-hydroxybutyrate metabolism pathways involving AKR7A2, ABAT, and SSADH.

**a**

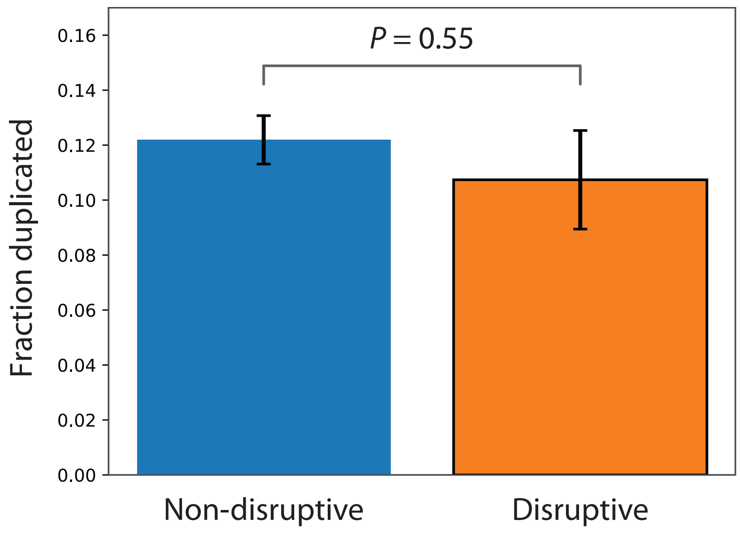


**b**

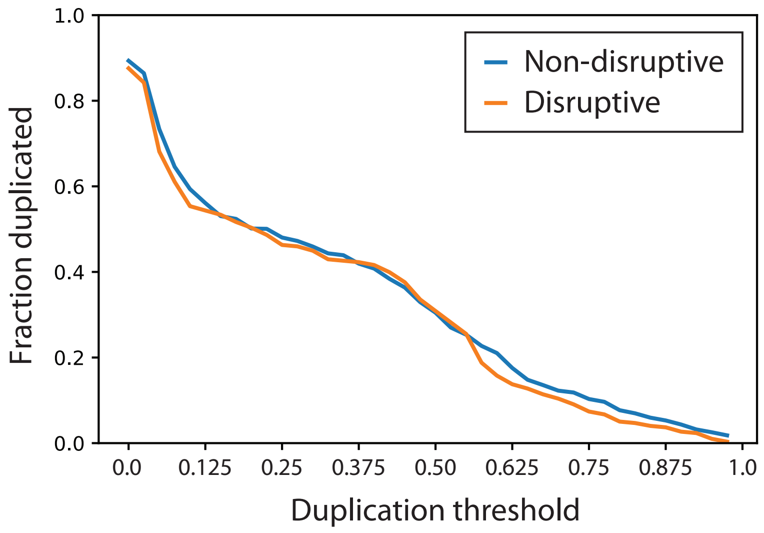


**Supplementary Figure 3**. Disruptive variants are not biased towards redundant genes. (**a**) Genes harboring non-disruptive and disruptive variants were intersected with genes found in the Duplicated Genes Database. For non-disruptive and disruptive variants, the fraction of genes that overlap with genes listed in this database were plotted. Error bars indicate ±SE of proportion. *P* values by two-tailed *Z*-test. (**b**) Sets of sequence-conserved, functionally similar proteins were generated at increasingly stringent thresholds for defining gene duplication. Proteins harboring non-disruptive and disruptive variants were intersected with these sets and the fraction of duplicate proteins was plotted at different duplication thresholds. A higher duplication threshold indicates a more stringent cutoff criteria for defining functionally similar proteins.

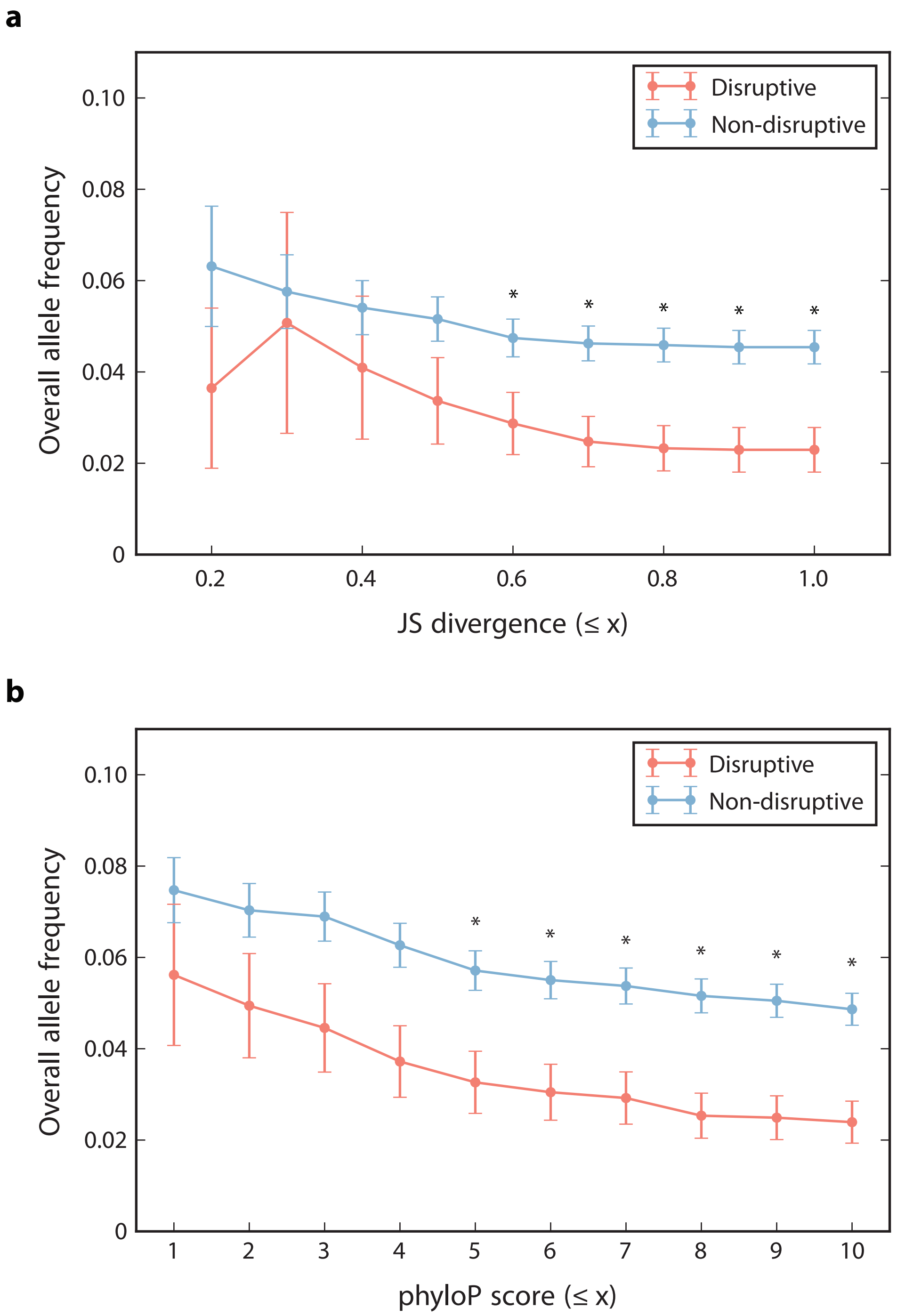


**Supplementary Figure 4**. Purifying selection may be stronger for disruptive variants at conserved protein sites. (**a**) Relationship between conservation and allele frequency for disruptive and non-disruptive variants examined across increasingly stringent cutoffs for JS divergence scores. Error bars indicate ±SE of mean. (**b**) Relationship between conservation and overall allele frequency for disruptive and non-disruptive variants examined across increasingly stringent phyloP scores. Error bars indicate ±SE of mean. *P* values by one-tailed Z-test. * $P < 0.05$.

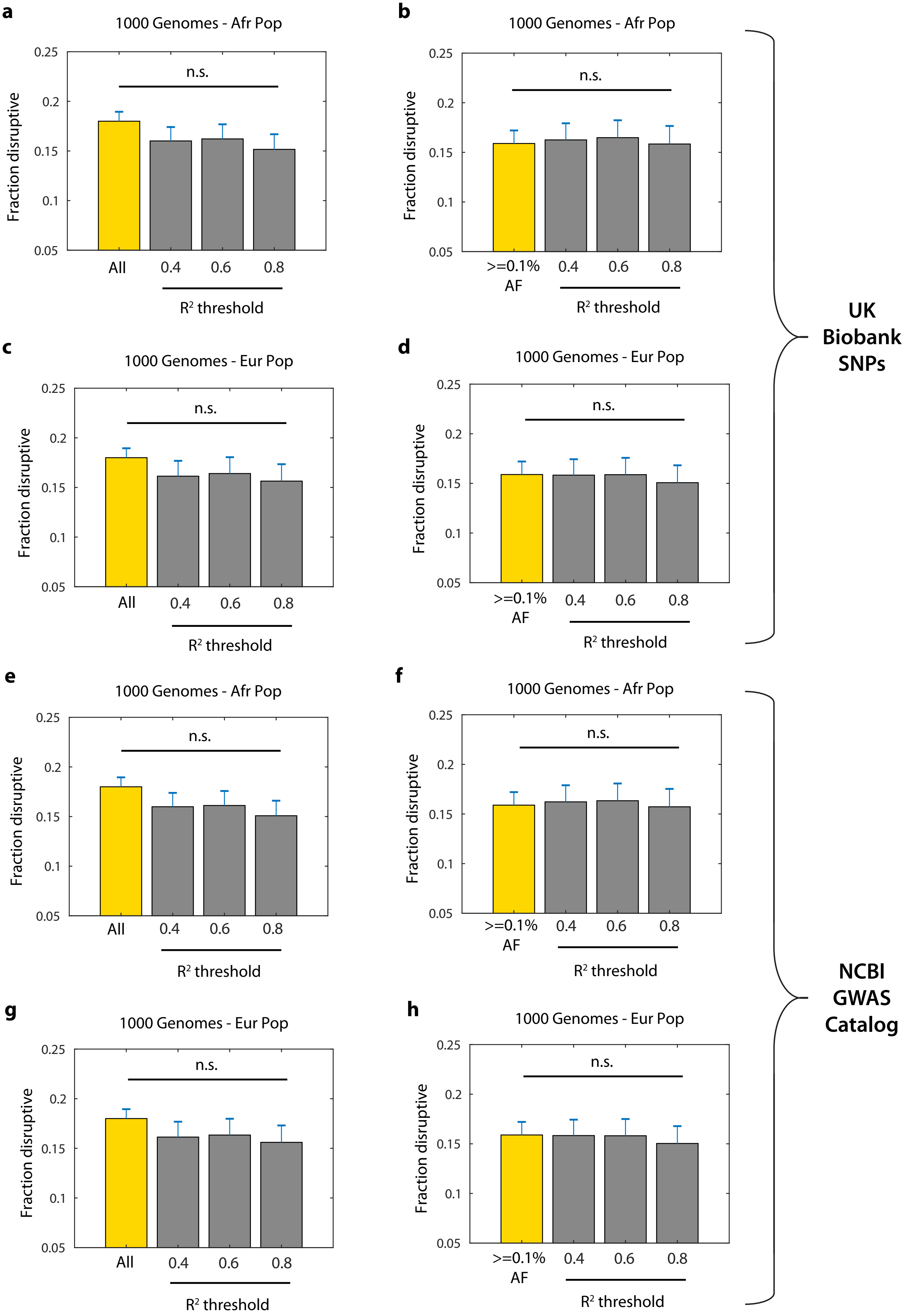

a
1000 Genomes - Afr Pop
Fraction disruptive
0.25
0.2
0.15
0.1
0.05
n.s.
All
0.4
0.6
0.8
R² threshold
b
1000 Genomes - Afr Pop
Fraction disruptive
0.25
0.2
0.15
0.1
0.05
n.s.
>=0.1%
AF
0.4
0.6
0.8
R² threshold
c
1000 Genomes - Eur Pop
Fraction disruptive
0.25
0.2
0.15
0.1
0.05
n.s.
All
0.4
0.6
0.8
R² threshold
d
1000 Genomes - Eur Pop
Fraction disruptive
0.25
0.2
0.15
0.1
0.05
n.s.
>=0.1%
AF
0.4
0.6
0.8
R² threshold
UK
Biobank
SNPs
e
1000 Genomes - Afr Pop
Fraction disruptive
0.25
0.2
0.15
0.1
0.05
n.s.
All
0.4
0.6
0.8
R² threshold
f
1000 Genomes - Afr Pop
Fraction disruptive
0.25
0.2
0.15
0.1
0.05
n.s.
>=0.1%
AF
0.4
0.6
0.8
R² threshold
g
1000 Genomes - Eur Pop
Fraction disruptive
0.25
0.2
0.15
0.1
0.05
n.s.
All
0.4
0.6
0.8
R² threshold
h
1000 Genomes - Eur Pop
Fraction disruptive
0.25
0.2
0.15
0.1
0.05
n.s.
>=0.1%
AF
0.4
0.6
0.8
R² threshold
NCBI
GWAS
Catalog

**Supplementary Figure 5**. Disruptive variants show no bias towards GWAS phenotypes. (**a**) Fraction of disruptive variants for variants found in the 1000 Genomes phase 3 Afr population at all allele frequencies is plotted (yellow). Fraction of disruptive variants for variants for variants in LD with GWAS SNPs listed in the UK biobank at $R^2$ thresholds of ≥ 0.4, ≥ 0.6, and ≥ 0.8 are also plotted (grey). (**b**) Fraction of disruptive variants for variants found in the 1000 Genomes phase 3 Afr population at AF ≥ 0.1% is plotted (yellow). Fraction of disruptive variants for variants for variants in LD with GWAS SNPs listed in the UK biobank at $R^2$ thresholds of ≥ 0.4, ≥ 0.6, and ≥ 0.8 are also plotted (grey). (**c**) Same analysis as (a) but restricted to 1000 Genomes phase 3 Eur population. (**d**) Same analysis as (b) but restricted to 1000 Genomes phase 3 Eur population. (**e-h**) Same analyses as (a-d) but for GWAS SNPs listed in the NCBI GWAS Catalog. Error bars indicate +SE of proportion. *P* values by two-tailed *Z*-test. n.s. = not significant.

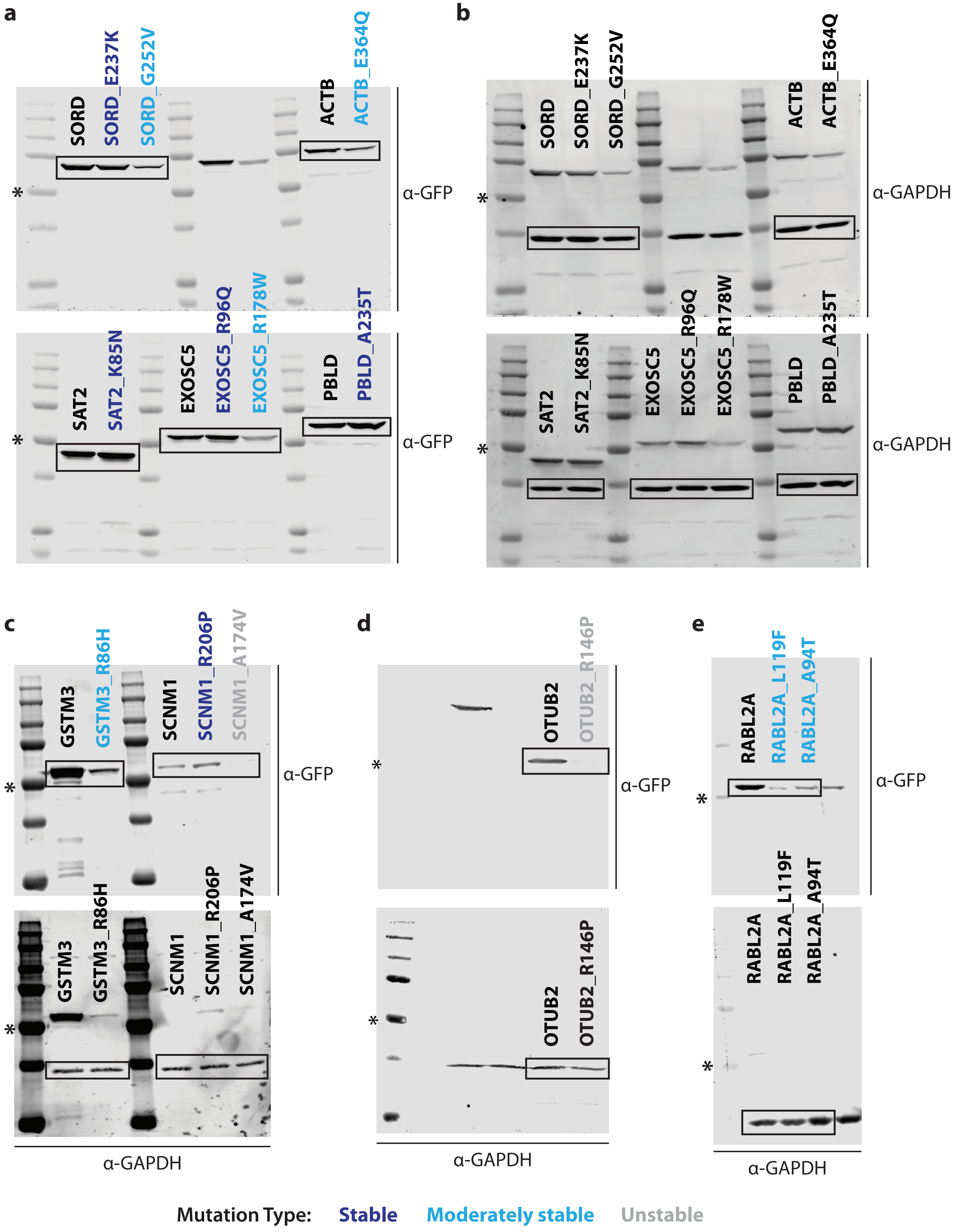
a
SORD
SORD_E237K
SORD_G252V
ACTB
ACTB_E364Q
α-GFP
SAT2
SAT2_K85N
EXOSC5
EXOSC5_R96Q
EXOSC5_R178W
PBLD
PBLD_A235T
α-GFP
b
SORD
SORD_E237K
SORD_G252V
ACTB
ACTB_E364Q
α-GAPDH
SAT2
SAT2_K85N
EXOSC5
EXOSC5_R96Q
EXOSC5_R178W
PBLD
PBLD_A235T
α-GAPDH
c
GSTM3
GSTM3_R86H
SCNM1
SCNM1_R206P
SCNM1_A174V
α-GFP
GSTM3
GSTM3_R86H
SCNM1
SCNM1_R206P
SCNM1_A174V
α-GAPDH
d
OTUB2
OTUB2_R146P
α-GFP
OTUB2
OTUB2_R146P
α-GAPDH
e
RABL2A
RABL2A_L119F
RABL2A_A94T
α-GFP
RABL2A
RABL2A_L119F
RABL2A_A94T
α-GAPDH
Mutation Type: Stable Moderately stable Unstable

**Supplementary Figure 6**. Uncropped Western blots for stable, moderately stable, and unstable GFP expression examples in Fig. 3a. (**a**) Westerns for wild-type and corresponding mutant proteins detected by α-GFP. (**b**) α-GAPDH controls for westerns for wild-type and corresponding mutant proteins detected in (a). (**c**) *Upper*: Westerns for wild-type and corresponding mutant proteins detected by α-GFP. *Lower*: α-GAPDH controls for western in upper. (**d**) *Upper*: Westerns for wild-type and corresponding mutant proteins detected by α-GFP. 50 kDa marker assigned using ladder from lower. *Lower*: α-GAPDH controls for western in upper. (**e**) *Upper*: Westerns for wild-type and corresponding mutant proteins detected by α-GFP. *Lower*: α-GAPDH controls for western in upper. In (a-e), stable, partially stable, and unstable mutations are labeled in blue, cyan, and gray, respectively; all α-GAPDH controls were detected using stripped membranes. Bands unrelated to this project are not boxed and were not used in any analyses. Bands corresponding to α-GFP and α-GAPDH examples used in Fig. 3a are enclosed in black boxes.
* indicates 50 kDa marker.

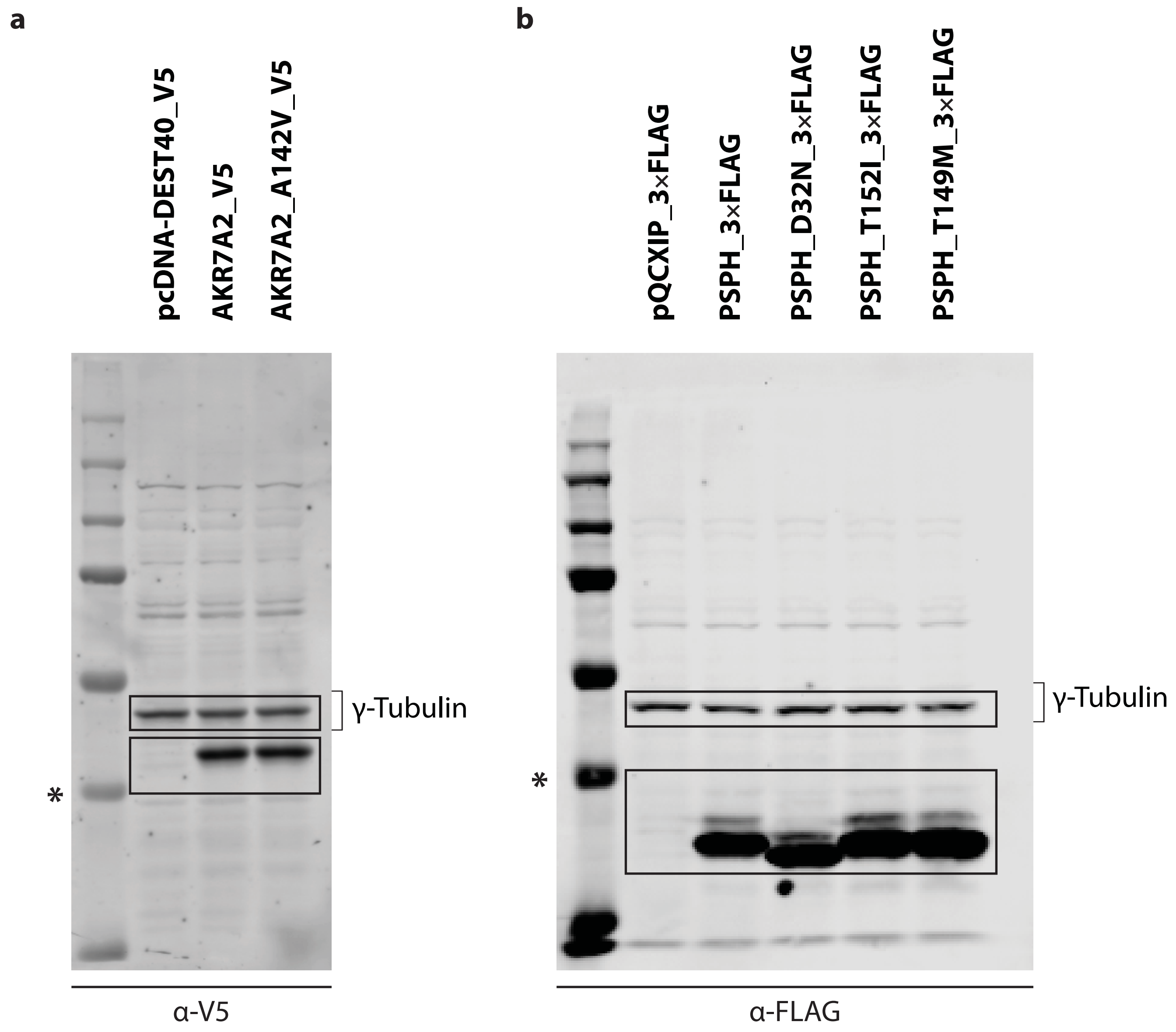


**Supplementary Figure 7**. Uncropped Western blots for AKR7A2 and PSPH mutant proteins. (**a**) Westerns for wild-type and A142T variant of AKR7A2 detected by α-V5. α-γ-Tubulin control ran on an unstripped membrane. (**b**) Westerns for wild-type and mutant PSPH proteins detected by α-FLAG. α-γ-Tubulin control ran on an unstripped membrane. In (a) and (b), black boxes indicate where figures were cropped for Western blots in Fig. 3e and Fig. 5a, respectively. * indicates 37 kDa marker.

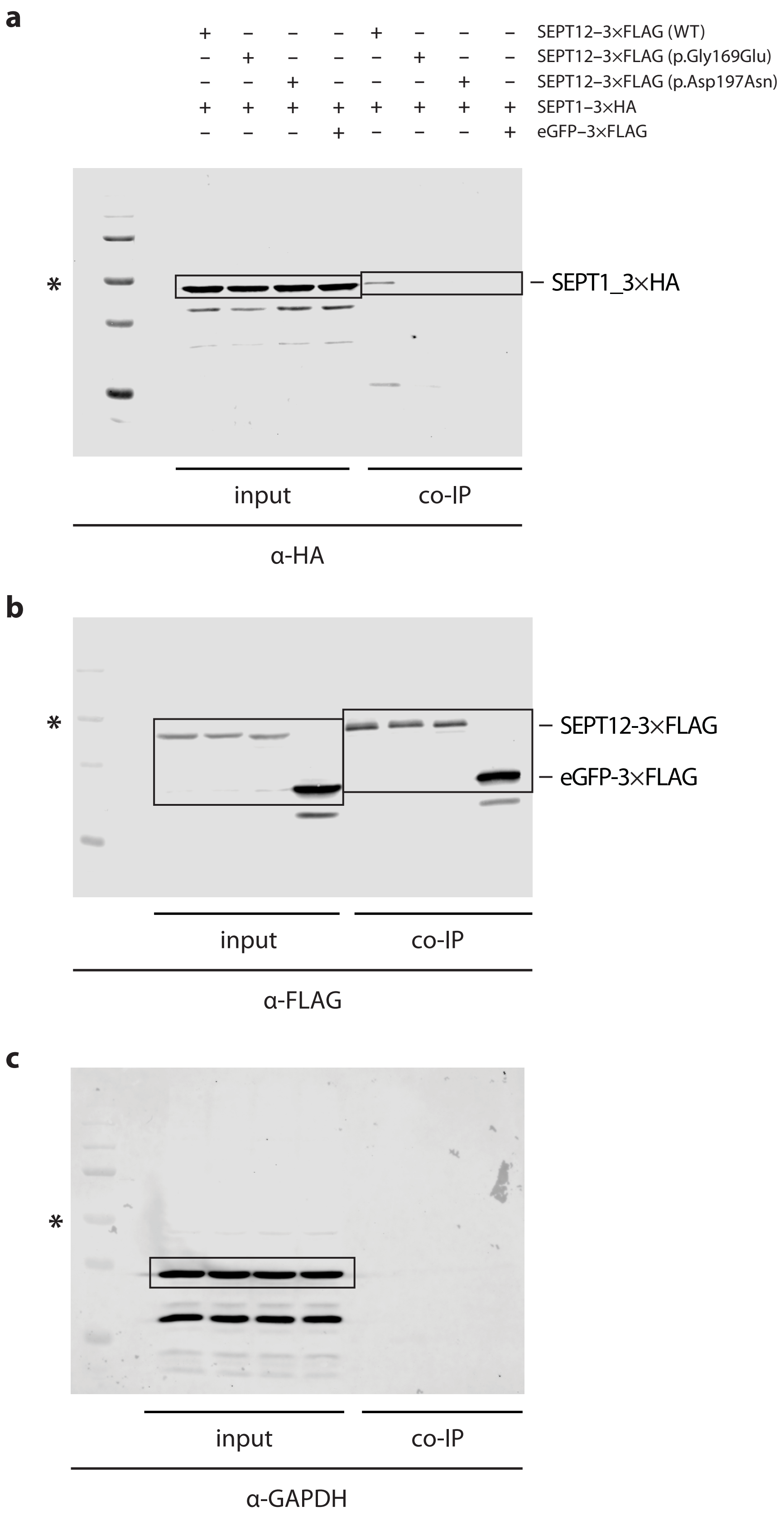


**Supplementary Figure 8**. Uncropped Western blots for SEPT12-SEPT1 co-IPs. (**a**) Westerns for SEPT12 co-IP of SEPT1 using α-FLAG beads. SEPT1 is tagged with 3×HA and detected using α-HA. (**b**) Westerns for SEPT12 co-IP of SEPT1 using α-FLAG beads. SEPT12 is tagged with 3×FLAG and detected using α-FLAG. eGFP tagged with 3×FLAG is included as a negative co-IP control. (**c**) α-GAPDH control for SEPT12-SEPT1 co-IP ran on a stripped membrane. In (a-c), black boxes indicate where figures were cropped for Western blot in Fig. 5e. * indicates 50 kDa marker.

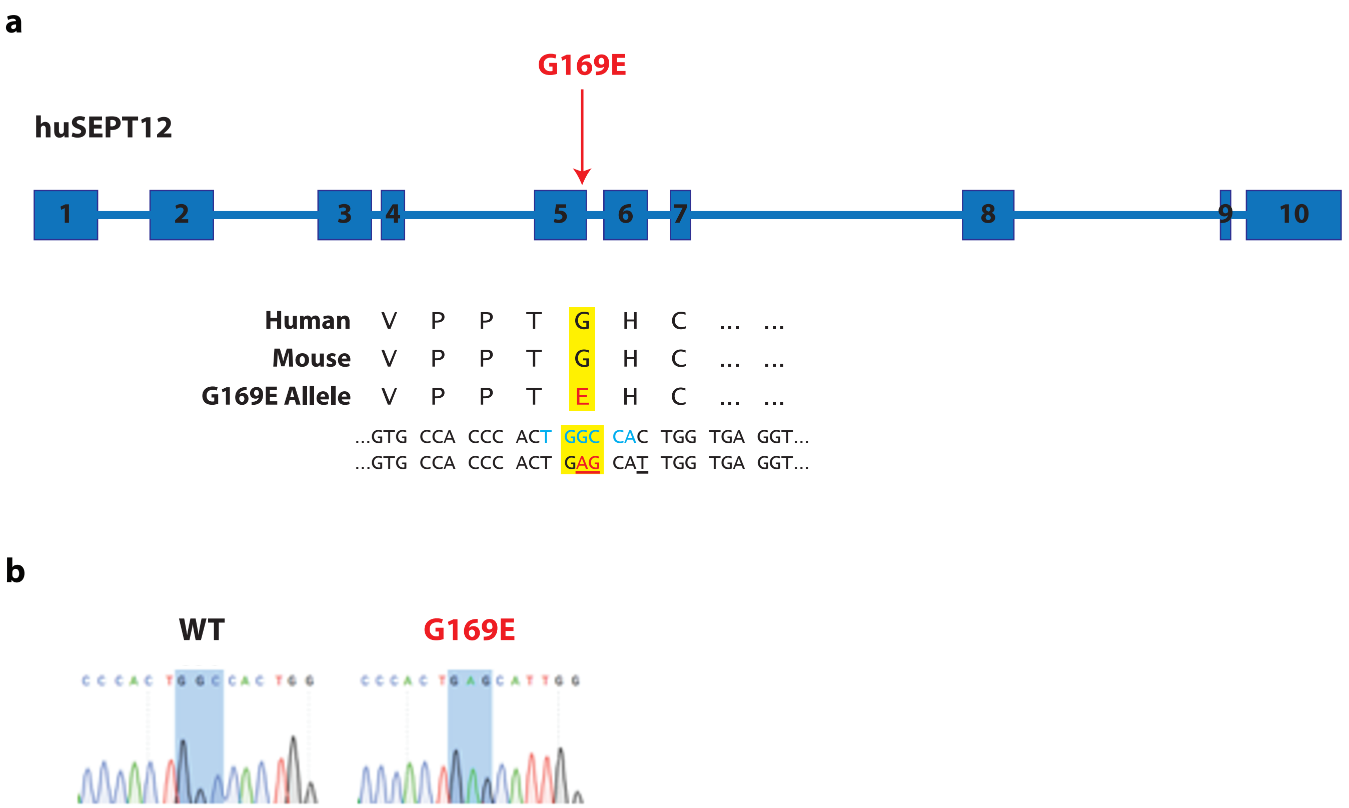


**Supplementary Figure 9**. Diagramming and validating CRISPR/Cas9 genome editing for generating *Sept12* G169E mutant mice. (a) Schematic of human *SEPT12* and approximate location of desired variant. Below schematic are the amino acid and nucleotide sequences of region where targeted allele resides in human and mouse genomes, as well as the point mutations introduced by homologous-directed repair with a synthetic single-stranded oligonucleotide template. Yellow highlight = residue/codon positions of interest, red text/underline = nucleotide changes to generate G169E variant; black text/underline = silent nucleotide change to prevent Cas9 re-cleavage; blue text = restriction enzyme site used for genotyping. (b) Chromatograms for WT and $Sept12^{G169E/G169E}$ mice.

## Missense mutations per individual

| Allele Frequency Range | Nonsynonymous Variants | | Stop-loss Variants | | Stop-gain Variants | | Missense mutations | |
|---|---|---|---|---|---|---|---|---|
| | Min | Max | Min | Max | Min | Max | Range | Average |
| < 0.5% | 224 | 806 | 1.1 | 1.7 | 3.9 | 10 | 219-794.3 | 506.7 |
| 0.5 - 5.0% | 540 | 2377 | 1.1 | 4.3 | 5.3 | 19 | 533.6-2353.7 | 1443.7 |
| > 5.0% | 10056 | 11198 | 37 | 40 | 24 | 28 | 9995-11130 | 10562.5 |
| | | | | | | **Total number of missense mutations per individual** | **10748-14278** | **12513** |

## Functional mutations per individual

| Allele Frequency Range | Nonsynonymous Variants | | Stop-loss Variants | | Stop-gain Variants | | Missense mutations | |
|---|---|---|---|---|---|---|---|---|
| | Min | Max | Min | Max | Min | Max | Range | Average |
| < 0.5% | 76 | 190 | 0.81 | 1.1 | 3.4 | 7.5 | 71.8-181.4 | 126.6 |
| 0.5 - 5.0% | 77 | 130 | 0.8 | 1 | 3.8 | 11 | 72.4-118 | 95.2 |
| > 5.0% | not reported | | | | | | | |
| | | | | | | **Total number of functional missense mutations per individual** | **144-299** | **222** |
| | | | | | | **Disruption rate per individual** | **1.34-2.10%** | **1.77%** |

**Supplementary Table 1. Calculation of Functional Missense Mutations for 1000 Genomes Project Phase I**

*Citation* : The 1000 Genomes Project Consortium. An integrated map of genetic variation from 1,092 human genomes. Nature 491, 56-65 (2012).

*Calculation notes* : Missense mutations were obtained by subtracting stop-loss and stop-gain variants from non-synonymous variants across allele frequency ranges.

**Functional missense mutations per individual**

| Allele Frequency Range | Nonsynonymous Variants | Stop-loss Variants | Stop-gain Variants | Missense Variants | Functional Mutations | |
|---|---|---|---|---|---|---|
| | | | | | GERP > 2.0 | No filter |
| < 0.5% | 165 | 0 | not reported | 165 | 32 | 41 |
| 0.5 - 5.0% | 474 | 0 | | 474 | 58 | 81 |
| > 5.0% | 6404 | 0.5 | | 6403.5 | 394 | 742 |
| **Sum** | 7043 | 0.5 | | **7042.5** | **484** | **864** |
| | | | **Disruption rate per individual** | | **6.87%** | **12.27%** |

**Supplementary Table 2. Calculation of Functional Missense Mutations for GoNL**

*Citation* : The Genome of the Netherlands Consortium. Whole-genome sequence variation, population structure and demographic history of the Dutch population. Nat Genet 46, 818-825 (2014).
*Calculation notes* : Missense mutations were obtained by subtracting stop-loss and stop-gain variants from non-synonymous variants across allele frequency ranges.

**Functional mutations per individual**

| Allele Frequency Range | Missense Variants | Nonsense Variants | Nonsynonymous Variants | Functional Mutations | |
|---|---|---|---|---|---|
| | | | | Conservative | Liberal |
| All alleles | 5754 | 35 | **5789** | **318** | **580** |
| | | | **Disruption rate per individual** | **5.49%** | **10.02%** |

**Supplementary Table 3. Calculation of Functional Nonsynonymous Mutations for ESP Phase I**

*Citation* : Tennessen, J.A., et al. Evolution and Functional Impact of Rare Coding Variation from Deep Sequencing of Human Exomes. Science 337, 64-69 (2012).

*Calculation notes* : Functional mutations by mutation type were not reported in citation. As such, functional nonsynonymous mutations, including nonsense variants, are reported. Disruption rate per individual is therefore a small overestimate of the number of functional missense mutations per individual.

**Supplementary Note 1. Selection of Y2H protein-protein interaction pairs from a reference interactome.**

To select interaction partners for each mutant protein tested in our SNV-perturbation screen, we first leveraged a Y2H reference interactome comprised of over 14,000 known wild-type protein-protein interactions reported in four manuscripts[1-4]. Since these published interactions are retestable by our version of Y2H, we only tested SNVs that corresponded with protein-protein interactions from this reference interactome. This requirement for retestable wild-type interactions found in the literature-reported reference interactome dramatically reduces the search space in which we probe for disruptive SNVs and prevented the need for an all-by-all Y2H interaction screen.

On average, each protein in the reference interactome has between 2-3 interaction partners. We note that we tested 847 unique genes against 2,185 corresponding interaction partners (~2.5 interaction partners per gene-encoded protein). For each wild-type protein-protein interaction, we then tested whether corresponding SNVs for each interaction can perturb that interaction. This consisted of 2,009 SNVs found on 847 unique genes. Since each of these genes has ~2.5 interaction partners, this results in a total of 4,797 SNV-interaction pairs tested (**Fig. 1c**).

We further note that Y2H has been extensively demonstrated to detect biologically meaningful interactions across many organisms in many studies[1,3-8]. To further confirm the biological significance of the interactions used in this study, we examined the co-expression of protein abundance levels corresponding to interactions used in our study. Using protein expression levels for 30 adult and fetal tissues and cell types from the Human Proteome Map[9], we found that proteins corresponding to interactions used in our study were significantly more likely to be co-expressed than random protein pairs, confirming the *in vivo* biological significance of the interactions used in the study (**Supplementary Figure 1f**).

**Supplementary Note 2. Calculating the fraction of disruptive missense variants per individual.**

We note that allele counts in ExAC correspond predominantly to common alleles (MAF > 1%). Therefore the fraction of disruptive alleles that are common will have the greatest influence on the average number of interaction-disruptive variants per individual. Disruptive and total allele counts corresponding to **Fig. 2b** are presented below in **Supplementary Table 4**.

| MAF bins | <0.1% | 0.1-1.0% | 1-10% | >10% | Cumulative |
|---|---|---|---|---|---|
| Disruptive alleles | 173 | 70 | 35 | 20 | 298 |
| Total alleles | 865 | 390 | 213 | 208 | 1676 |
| Disruption rate | 20.0% | 17.9% | 16.4% | 9.6% | 17.8% |

**Supplementary Table 4:** Disruption rate for tested ExAC variants calculated across four MAF bins.

Next, we calculated the site frequency spectrum for ExAC alleles annotated as *missense_variant* in at least one transcript by summing the adjusted overall allele counts (listed as *AC_adj* in the ExAC database) per MAF bin and dividing each bin count by the total adjusted overall allele count across all bins as shown in the formula below:

$$f_i = \frac{Allele\ count_i}{\sum_i^4 Allele\ count_i} \quad (1)$$

where $i$ represents the four MAF bins examined and $f_i$ represents the fraction of missense variants per individual expected to be in MAF bin $i$. Applying this equation to all four MAF bins yields the following per-individual proportions:

| MAF bins | <0.1% | 0.1-1.0% | 1-10% | >10% |
|---|---|---|---|---|
| Mean proportion of missense SNVs ($f_i$) | 0.0173 | 0.0254 | 0.0793 | 0.8780 |
| Adjusted disruption rate | 0.00346 | 0.00456 | 0.0130 | 0.0844 |

**Supplementary Table 5:** Mean proportion of missense SNVs per individual across four MAF bins.

As noted earlier, most variants per individual genome, 87.8%, are very common (MAF > 10%). The *Adjusted disruption rate* per MAF bin listed in **Supplementary Table 5** was obtained by multiplying the *Mean proportion of missense SNVs* by the *Disruption rate* listed in **Supplementary Table 4**. Summing the *Adjusted disruption rate* across all MAF bins yields a *mean disruption rate per individual* = 10.5% ± 1.8% as reported in **Fig. 2c**, where the error is calculated by the delta method.

**Supplementary Note 3. Categorizing stable, moderately stable, and unstable mutant proteins.**

Plate reader raw data from each 96-well plate consists of two fluorescence readings corresponding to GFP and mCherry expression in each well for proteins expressed in pDEST-DUAL vector. Wildtype/mutant groups are segregated to be on the same plate so that they can be processed together. Each plate is allocated eight wells for background controls: four wells transfected with empty pDEST-DUAL vector such that only mCherry expression is expected, used as a GFP baseline, and four wells transfected with empty pcDNA-DEST47 vector where no GFP or mCherry expression is expected, used as a mCherry baseline. All expression values are normalized as a z-score representing the number of standard deviations away from the mean background expression.

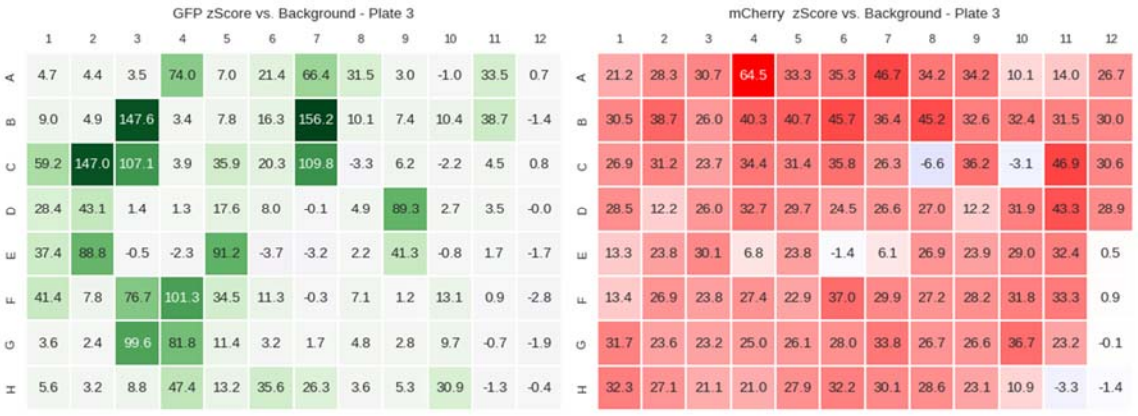


**Supplementary Figure 10:** All fluorescence readings are represented as a z-score away from the controls in that plate's 12th column. A12-D12 serve as GFP background using empty pDEST-DUAL vector, E12-H12 serve as mCherry background using empty pcDNA-DEST47 vector.

Next, we apply basic quality control filters. A fluorescence reading is considered significant if the *P* value associated with its z-score on the background normal distribution is less than 0.05. We only perform analysis on experiments with significant wildtype expression for both GFP and mCherry channels. Further, we filter out any mutants that do not present significant mCherry expression.

We calculate wildtype activation and fold change to determine whether a mutant well is under-expressing GFP relative to its corresponding wildtype. Wildtype activation is the ratio between the GFP z-score and the mCherry z-score in the wildtype well for that ORF and is reported as "Wildtype stability score" in **Fig. 3b**. Similarly, mutant activation is the ratio between the GFP z-score and mCherry z-score in the mutant well for an ORF and is reported as "Mutant stability score" in **Fig. 3b**. We then calculated fold change as the ratio of mutant activation over wildtype activation, reported in **Fig. 3d** as the "stability score ratio." All values are reported in **Supplementary Data 5**.

$$\text{WT Activation} = \frac{\text{Z}(\text{GFP}_{\text{WT}})}{\text{Z}(\text{mCherry}_{\text{WT}})} \tag{2}$$

$$\text{Fold Change} = \frac{\frac{Z(GFP_{Mut})}{Z(mCherry_{Mut})}}{\frac{Z(GFP_{WT})}{Z(mCherry_{WT})}} \tag{3}$$

As an added quality control step, experiments with WT Activation less than 1.0 are removed. All other experiments are then classified into three groups: *stable* if the fold change is above 0.5, *moderately stable* if the fold change is between 0.5 and 0.0, and *unstable* if the fold change is less than 0.0.

**Supplementary Note 4. Dissecting the impact of variants in disease.**

Studying complex disease is difficult since very often no single variant alone is fully penetrant. Nonetheless, the simplest case of complex disease, digenic inheritance in which two genes both contribute to a single phenotype, is actually quite prevalent. Searching HGMD[10] yields a total of 365 mutations that contribute to digenic inheritance. Moreover, a search through PubMed for "digenic mutations" yielded 378 papers, although this strictly refers to cases in which two heterozygous mutations in different genes must occur together for the disease phenotype to manifest. Cases in which the impact of a disease-causing mutation in one gene is influenced by a polymorphic variant in another gene are far more common and are extensively documented in HGMD. Such variants are often only partially penetrant, resulting in disease in only particular genetic backgrounds[11]. While dissecting how these variants modulate each other's impact is not straightforward, individually assessing the impact of these variants in isolation is a crucial first step towards understanding how these variants function epistatically. In this context, our study represents an important resource for examining what fraction of population variants are functional and could conceivably play a role in disease risk as a result.

**Supplementary Note 5. Examining the potential drug-relevance of disruptive SNVs**

The results of our study may have important implications in related fields such as pharmacogenomics and toxicogenomics. Disruptive SNVs on enzymes may alter the metabolic kinetics of impacted enzymes, while SNVs on transporters and targets of drugs may lead to changes in the pharmacokinetic and pharmacodynamic properties of their corresponding proteins. For example, the D816H/V mutations on the receptor tyrosine kinase, KIT, confers resistance to imatinib and sunitinib by shifting the conformational equilibrium of KIT[12]. To explore the potential relevance of our SNV disruption data, we generated a dataset of disruptive SNVs potentially relevant to pharmacogenomics and toxicogenomics by intersecting our SNVs disruption dataset with four sets of genes: all human enzymes, drug-metabolizing enzymes, drug targets, and drug transporters. The list of all human enzyme genes was obtained from HumanCyc version 21.5[13], while the lists of drug-related genes were obtained from DrugBank version 5.1.2[14]. Among the SNVs that we tested, 350 were on enzymes, and 84 of them disrupted at least one interaction. A table consisting of all disruptive SNVs that may be relevant to drug action is provided in **Supplementary Data 7**.

**Supplementary Note 6. Background and motivation for Protein Complementation Assay.**

Protein Complementation Assay (PCA) is a protein-protein interaction assay performed in HEK 293T cells in which a bait and prey protein are fused to two complementary fragments of a fluorescent protein, YFP. If the bait and prey protein successfully interact, the two YFP fragments will stably bind and fluoresce as a result. PCA is a particularly valuable assay in protein-protein interaction screens because it is high-throughput, and it provides an independent assay for validating the quality of protein-protein interactions detected through Y2H screens. For this reason, PCA is commonly used in many interactome screens, including in *Arabidopsis*[7], yeast[3,8], and human[4,15]. Notably, PCA can also be used to validate that two proteins do not interact, which is important when testing the impact of disruptive variants. To do this, loss of fluorescence signal in PCA for mutant interaction pairs relative to wild-type pairs is measured to validate that Y2H-tested mutations are indeed disruptive[16,17].

**Supplementary References**


1. Rual, J.-F.*, et al.* Towards a proteome-scale map of the human protein-protein interaction network. *Nature* **437**, 1173-1178 (2005).
2. Venkatesan, K.*, et al.* An empirical framework for binary interactome mapping. *Nat. Methods* **6**, 83-90 (2009).
3. Yu, H.*, et al.* High-Quality Binary Protein Interaction Map of the Yeast Interactome Network. *Science* **322**, 104-110 (2008).
4. Rolland, T.*, et al.* A proteome-scale map of the human interactome network. *Cell* **159**, 1212-1226 (2014).
5. Walhout, A.J.M.*, et al.* Protein Interaction Mapping in *C. elegans* Using Proteins Involved in Vulval Development. *Science* **287**, 116-122 (2000).
6. Boulton, S.J.*, et al.* Combined Functional Genomic Maps of the *C. elegans* DNA Damage Response. *Science* **295**, 127-131 (2002).
7. *Arabidopsis* Interactome Mapping Consortium. Evidence for Network Evolution in an *Arabidopsis* Interactome Map. *Science* **333**, 601-607 (2011).
8. Vo, T.V.*, et al.* A proteome-wide fission yeast interactome reveals network evolution principles from yeasts to human. *Cell* **164**, 310-323 (2016).
9. Kim, M.-S.*, et al.* A draft map of the human proteome. *Nature* **509**, 575 (2014).
10. Stenson, P.D.*, et al.* The Human Gene Mutation Database: towards a comprehensive repository of inherited mutation data for medical research, genetic diagnosis and next-generation sequencing studies. *Hum. Genet.* **136**, 665-677 (2017).
11. Cooper, D.N., Krawczak, M., Polychronakos, C., Tyler-Smith, C. & Kehrer-Sawatzki, H. Where genotype is not predictive of phenotype: towards an understanding of the molecular basis of reduced penetrance in human inherited disease. *Hum. Genet.* **132**, 1077-1130 (2013).
12. Gajiwala, K.S.*, et al.* KIT kinase mutants show unique mechanisms of drug resistance to imatinib and sunitinib in gastrointestinal stromal tumor patients. *Proc. Natl. Acad. Sci. U.S.A.* **106**, 1542-1547 (2009).
13. Romero, P.*, et al.* Computational prediction of human metabolic pathways from the complete human genome. *Genome Biol.* **6**, R2 (2004).
14. Wishart, D.S.*, et al.* DrugBank 5.0: a major update to the DrugBank database for 2018. *Nucleic Acids Res.* **46**, D1074-D1082 (2018).
15. Yang, X.*, et al.* Widespread Expansion of Protein Interaction Capabilities by Alternative Splicing. *Cell* **164**, 805-817 (2016).
16. Sahni, N.*, et al.* Widespread macromolecular interaction perturbations in human genetic disorders. *Cell* **161**, 647-660 (2015).
17. Yi, S., Liu, N.-N., Hu, L., Wang, H. & Sahni, N. Base-resolution stratification of cancer mutations using functional variomics. *Nat. Protoc.* **12**, 2323 (2017).
