# Supplementary material for: Extensive disruption of protein interactions by genetic variants across the allele frequency spectrum in human populations
Source: Nat Commun. 2019 Sep 12;10:4141. doi: 10.1038/s41467-019-11959-3 (PMC6742646; doi:10.1038/s41467-019-11959-3)
Supplement: Supplementary file 3 — Reporting Summary [file 41467_2019_11959_MOESM3_ESM.pdf]

nature research

Corresponding author(s): Haiyuan Yu

Last updated by author(s): Jul 24, 2019

Reporting Summary

Nature Research wishes to improve the reproducibility of the work that we publish. This form provides structure for consistency and transparency in reporting. For further information on Nature Research policies, see Authors & Referees and the Editorial Policy Checklist.

Statistics

For all statistical analyses, confirm that the following items are present in the figure legend, table legend, main text, or Methods section.

n/a	Confirmed	
☐	☒	The exact sample size (*n*) for each experimental group/condition, given as a discrete number and unit of measurement
☐	☒	A statement on whether measurements were taken from distinct samples or whether the same sample was measured repeatedly
☐	☒	The statistical test(s) used AND whether they are one- or two-sided<br>*Only common tests should be described solely by name; describe more complex techniques in the Methods section.*
☒	☐	A description of all covariates tested
☐	☒	A description of any assumptions or corrections, such as tests of normality and adjustment for multiple comparisons
☐	☒	A full description of the statistical parameters including central tendency (e.g. means) or other basic estimates (e.g. regression coefficient) AND variation (e.g. standard deviation) or associated estimates of uncertainty (e.g. confidence intervals)
☐	☒	For null hypothesis testing, the test statistic (e.g. *F*, *t*, *r*) with confidence intervals, effect sizes, degrees of freedom and *P* value noted<br>*Give P values as exact values whenever suitable.*
☒	☐	For Bayesian analysis, information on the choice of priors and Markov chain Monte Carlo settings
☒	☐	For hierarchical and complex designs, identification of the appropriate level for tests and full reporting of outcomes
☒	☐	Estimates of effect sizes (e.g. Cohen's *d*, Pearson's *r*), indicating how they were calculated

Our web collection on statistics for biologists contains articles on many of the points above.

Software and code

Policy information about availability of computer code

Data collection	No software was used.
Data analysis	Statistical tests were performed using models built in Python (2.7).

For manuscripts utilizing custom algorithms or software that are central to the research but not yet described in published literature, software must be made available to editors/reviewers. We strongly encourage code deposition in a community repository (e.g. GitHub). See the Nature Research guidelines for submitting code & software for further information.

Data

Policy information about availability of data

All manuscripts must include a data availability statement. This statement should provide the following information, where applicable:

- Accession codes, unique identifiers, or web links for publicly available datasets
- A list of figures that have associated raw data
- A description of any restrictions on data availability

Clones for all mutations generated and analyzed in this study are listed in Supplementary Data 2-4 and are available upon request to Haiyuan Yu (haiyuan.yu@cornell.edu). Raw data for all Y2H-tested mutant interactions are also listed in Supplementary Data 2-4. DUAL-FLOU stability data for tested mutant proteins are listed in Supplementary Data 5. PCA interaction retest data is available in Supplementary Data 6. Allele counts and frequency data were obtained from ExAC version 0.3.1.

Field-specific reporting

Please select the one below that is the best fit for your research. If you are not sure, read the appropriate sections before making your selection.

☒ Life sciences ☐ Behavioural & social sciences ☐ Ecological, evolutionary & environmental sciences

For a reference copy of the document with all sections, see nature.com/documents/nr-reporting-summary-flat.pdf

Life sciences study design

All studies must disclose on these points even when the disclosure is negative.

Sample size	No statistical method was used to predetermine sample size. Sample sizes in high-thoughput experiments were determined by maximizing the total number of samples that could be analyzed in a particular experiment.
Data exclusions	No data were excluded from analyses.
Replication	Perturbed interactions had to be reproducible across three replicate screens to be reported as disruptive. Three biological replicates were performed for enzymatic experiments. Fertility tests were performed in two or more animals (exact sample counts are specified in figure legends).
Randomization	Protein interactions were selected in an unbiased manner for experimental test using yeast two-hybrid assay.
Blinding	Data blinding present through all experimental measurements. During measurements for high-throughput experiments, no knowledge of which particular mutation, its associated properties, or which interaction partners were being tested was made available.

Reporting for specific materials, systems and methods

We require information from authors about some types of materials, experimental systems and methods used in many studies. Here, indicate whether each material, system or method listed is relevant to your study. If you are not sure if a list item applies to your research, read the appropriate section before selecting a response.

Materials & experimental systems

n/a	Involved in the study	
☐	☒	Antibodies
☐	☒	Eukaryotic cell lines
☒	☐	Palaeontology
☐	☒	Animals and other organisms
☒	☐	Human research participants
☒	☐	Clinical data

Methods

n/a	Involved in the study	
☒	☐	ChIP-seq
☒	☐	Flow cytometry
☒	☐	MRI-based neuroimaging

Antibodies

Antibodies used	Anti-FLAG (Sigma, F1804) at 1:3000, anti-V5 (Invitrogen, R960-25) at 1:5000, anti-HA (Sigma, H3663) at 1:3000, anti-GFP (SCBT, sc-9996) at 1:1000, anti-GAPDH (Proteintech, 60004-1-Ig) at 1:3000, and anti-γ-Tubulin (Sigma, T5192) at 1:3000 dilutions were used for immunoblotting analyses.
Validation	Species validation of all primary antibodies used in this study can be found in the corresponding manufacturer´s websites.

Eukaryotic cell lines

Policy information about cell lines

Cell line source(s)	HEK293T cells were obtained from ATCC.
Authentication	Cell lines have been thoroughly tested and authenticated by ATCC.
Mycoplasma contamination	Cell lines were not tested for mycoplasma contamination.
Commonly misidentified lines (See ICLAC register)	No commonly misidentified cell lines were used.

Animals and other organisms

Policy information about studies involving animals; ARRIVE guidelines recommended for reporting animal research

Laboratory animals	F1 hybrids between strains FVB/NJ and B6(Cg)-Tyrc-2J/J of Mus musculus were used in CRISPR editing experiments. Wild-type, heterozygous, and homozygous males and females were bred to wild-type counterparts starting at 2 months until 7-15 months of age.
Wild animals	*Provide details on animals observed in or captured in the field; report species, sex and age where possible. Describe how animals were caught and transported and what happened to captive animals after the study (if killed, explain why and describe method; if released, say where and when) OR state that the study did not involve wild animals.*
Field-collected samples	*For laboratory work with field-collected samples, describe all relevant parameters such as housing, maintenance, temperature, photoperiod and end-of-experiment protocol OR state that the study did not involve samples collected from the field.*
Ethics oversight	All animal use was conducted under protocol (2004-0038) to J.C.S. and approved by Cornell University's Institutional Animal Use and Care Committee, affirming that we complied with all relevant ethical requirements for treatment and use of laboratory mice.

Note that full information on the approval of the study protocol must also be provided in the manuscript.
