# Supplementary material for: Extensive disruption of protein interactions by genetic variants across the allele frequency spectrum in human populations
Source: Nat Commun. 2019 Sep 12;10:4141. doi: 10.1038/s41467-019-11959-3 (PMC6742646; doi:10.1038/s41467-019-11959-3)
Supplement: Supplementary file 4 — Description of Additional Supplementary Files [file 41467_2019_11959_MOESM4_ESM.pdf]

Legends for Supplementary Data 1-7:

Title: Supplementary Data 1

Description: List of primer names and sequences used in high-throughput screens.

Title: Supplementary Data 2

Description: Interaction perturbation results for ExAC variants.

Title: Supplementary Data 3

Description: Interaction perturbation results for somatic mutations from COSMIC.

Title: Supplementary Data 4

Description: Interaction perturbation results for disease-associated mutations from HGMD.

Title: Supplementary Data 5

Description: DUAL-FLUO assay results for protein stability screen.

Title: Supplementary Data 6

Description: Protein Complementation Assay results for retested SNV-interaction pairs.

Title: Supplementary Data 7

Description: List of disruptive variants occurring in drug target-relevant genes.
